# Antiobesity Activity of *Elateriospermum tapos* Shell Extract in Obesity-Induced Sprague Dawley Rats

**DOI:** 10.3390/molecules26020321

**Published:** 2021-01-09

**Authors:** Kokila Vani Perumal, Nor Liyana Ja’afar, Che Norma Mat Taib, Nurul Husna Shafie, Hasnah Bahari

**Affiliations:** 1 Department of Human Anatomy, Universiti Putra Malaysia, Serdang 43400, Selangor, Malaysia; kokilavaniperumal@gmail.com (K.V.P.); norliyana1604@gmail.com (N.L.J.); chenorma@upm.edu.my (C.N.M.T.); 2 Department of Nutrition and Dietetics, Universiti Putra Malaysia, Serdang 43400, Selangor, Malaysia; nhusnashafie@upm.edu.my

**Keywords:** *Elateriospermum tapos*, obesity, cafeteria diet, high-fat diet

## Abstract

Obesity is one of the risk factors associated with cardiovascular diseases, hypertension, abnormal liver function, diabetes, and cancers. Orlistat is currently available to treat obesity, but it is associated with adverse side effects. Natural resources are widely used for obesity treatment. Hence, this study aimed to investigate the anti-obesity activity of *Elateriospermum tapos* (*E. tapos*) shell extract in obesity induced Sprague Dawley rats. The rats’ obesity was induced by a high-fat (HF) diet made up of 50% standard rat pellet, 20% milk powder, 6% corn starch, and 24% ghee and a cafeteria (CAF) diet such as chicken rolls, salty biscuits, cakes, and cheese snacks. A hot aqueous method for the extraction of *E. tapos* shells was applied by using 500 mL of distilled water for about 24 h. Various dosages of *E. tapos* shell extract (10 mg/kg, 100 mg/kg, and 200 mg/kg) were used. At the end of the study, body weight, caloric intake, organ weight, lipid profile, lipoprotein lipase (LPL) activity, and histopathology analysis were carried out. *E. tapos* shell extract treated groups showed a reduction in body weight, positive lipid-lowering effect, decrements in triglyceride accumulation and LPL activity, and positive improvement in histopathology analysis. A dose of 200 mg/kg showed the most effective result compared to 10 mg/kg and 100 mg/kg doses.

## 1. Introduction

Obesity is known as a condition of increased body mass index (BMI) and an excessive amount of body fat. It is associated with increased blood pressure, type 2 diabetes, cardiovascular diseases, and several types of cancer [1] such as liver, pancreas, colon, and rectum. According to the World Health Organization (WHO), BMI range is classified into normal (18.5 to 24.9 kg/m^2^), overweight (≥ 25 kg/m^2^), and obese (≥ 30 kg/m^2^), with severe obesity being defined by a BMI > 40 kg/m^2^. As a global health concern, the prevalence of overweight and obesity has increased drastically over the last few decades. In 2016, almost 650 million people were considered obese worldwide [2].

Orlistat is a common drug used to curb obesity. Correspondingly, it is used to lose around 5% to 10% of the body weight, but a regain of weight has been noticed when treatment is stopped. As a downfall, it is associated with side effects such as liver damage, diarrhea, nausea, dry mouth, fecal incontinence, flatulence, and steatorrhea [3]. Furthermore, Meridia (sibutramine) drug was withdrawn from the United States market after strong associations between the drug and several acute disorders such as heart failure and stroke [4]. Due to sparse efficacy in terms of current drug intervention, a novel alternative that directly targets obesity should be offered.

Natural resources are widely used to combat obesity due to being less toxic with fewer side effects [5]. One of them, *Elateriospermum tapos* (*E. tapos*), is widely distributed in the Southeast Asian tropical rain forest. In Malaysia, this species can be found in Jengka Forest Reserve, Peninsular Malaysia [6]. Several known phytochemicals were found in *E. tapos* shell including flavonoids, tannins, and alkaloids which hold therapeutic properties against many diseases [7]. Many studies have revealed that flavonoids have a significant effect on reducing body weight through the inhibition of the triglyceride level in the obese model. Flavonoids also played a pivotal part in the pathway of lipolysis in adipocytes [8]. Moreover, *E. tapos* shell has antioxidant properties that lower lipid peroxidation [9].

Previously, Perumal et al. conducted a study on the anti-obesity effect of *E. tapos* seed extract at different dosages of 5 mg/kg, 25 mg/kg, and 125 mg/kg in Sprague Dawley rats, whereby it positively affected the body weight, caloric intake, lipid profile, and histopathology [10]. As of today, no study has highlighted the potential role of *E. tapos* shell against obesity. Therefore, this study was conducted to study the antio-besity activity of *Elateriospermum tapos* (*E. tapos*) shell extract in obesity-induced Sprague Dawley rats.

## 2. Results

### 2.1. Effect of E. tapos Shell Extract on Body Weight and Caloric Intake

The rats fed with HF diet increased in body weight as compared to those fed with a normal diet. At the end of the study, the normal control (NC) group showed the lowest body weight and caloric intake as compared to the positive control (PC) group. The PC group showed the highest body weight and caloric intake followed by the DC group when compared to the NC group. There were significant differences (*p* < 0.05) in body weight at the death of TI and TIII as compared to the PC group. Whereas, the body weight of TII was lower than PC group. According to the results in Table 1, the caloric intake of PC and DC groups paralleled their body weight, while the *E. tapos* shell-treated groups (T I, T II, T III) did not correspond to their final weight.

### 2.2. Effect of E. tapos Shell Extract on Organ Weight

The NC group had the lightest organ weights (liver, RpWAT, visceral fat, and gonadal fat) as compared to the PC group. The PC group exhibited the heaviest organ weights (liver, RpWAT, visceral fat, and gonadal fat) compared to the NC group due to HF diet induction. All the *E. tapos* shell-treated groups (T I, T II, T III) had reduced organ weights (liver, RpWAT, visceral fat, and gonadal fat) as compared to the PC group. Furthermore, the DC group had significantly (*p* < 0.05) reduced deposition of adipose tissue as compared to the PC group (Table 2).

### 2.3. Effect of E. tapos Shell Extract on Lipid Profiles

As shown in Table 3, there were increments in TC and LDL and a reduction in HDL levels in the PC group as compared to the NC group. The DC group showed a decrease of the levels of TC and LDL and an increase in HDL as compared to the PC group. However, all the *E. tapos* shell-treated groups (T I, T II, T III) showed significant decrements (*p* < 0.05) in LDL levels and significant increments in HDL levels as compared to the PC group. 

### 2.4. Effect of E. tapos Shell Extract on Triglycerides

We observed an increase in the triglyceride deposition in the plasma, RpWAT, and liver from PC group when compared to the NC group. There was a decrease in triglyceride deposition in the plasma, RpWAT, and liver in the DC group as compared to the PC group. The triglyceride deposition in the plasma, RpWAT, and in the liver was reduced in all the *E. tapos* shell extract-treated groups (T I, T II, T III) as compared to the PC group (shown in Table 4). However, no significant difference was observed.

### 2.5. Effect of E. tapos Shell Extract on Lipoprotein Lipase (LPL) Activity

NC group demonstrated the lowest LPL activity as compared to the PC group. The LPL activity in the plasma and RpWAT showed a significant (*p* < 0.05) increase in the PC group as compared to the NC group. The lipoprotein lipase activity in the plasma and RpWAT in the DC group decreased as compared to the PC group. However, all the *E. tapos* shell extract-treated groups (T I, T II, T III) had reduced LPL activity in the plasma and RpWAT as compared to the PC group (Table 5).

### 2.6. LC–MS Analysis of E. tapos Shell Extract

**Figure 1:**
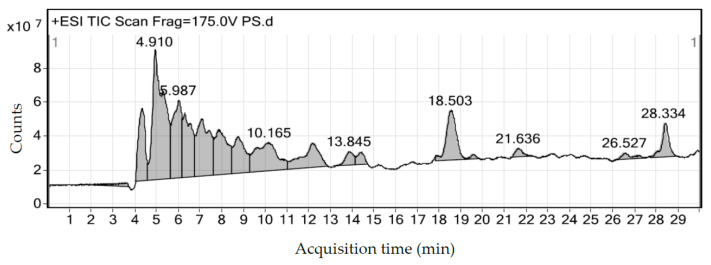
LC–MS analysis of *E. tapos* shell extract. The spectrum shows the compounds of *E. tapos* shell with retention time according to the chromatographic analysis.

The compounds were interpreted by the molecular weight by referring to the standard reference graphs. Table 6 showed the bioactive compound identified from the *E. tapos* shell extract. According to the chromatographic analysis of *E. tapos* shell extract in Figure 1, different peak were obtained at different retention time. The highest peak was 3′4′5-trimethoxyflavone is a flavonoid group with a retention time of 4.910 min (Figure 1). 

### 2.7. Effect of E. tapos Shell Extract on Histopathology of Liver

**Figure 2:**
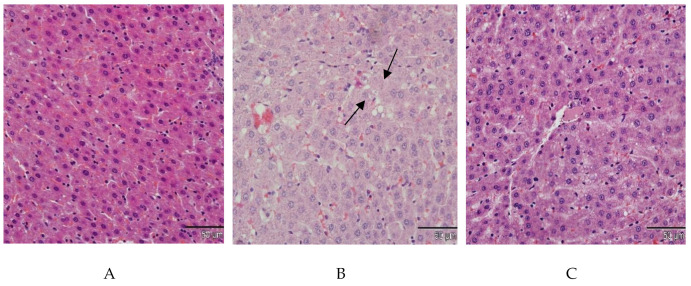

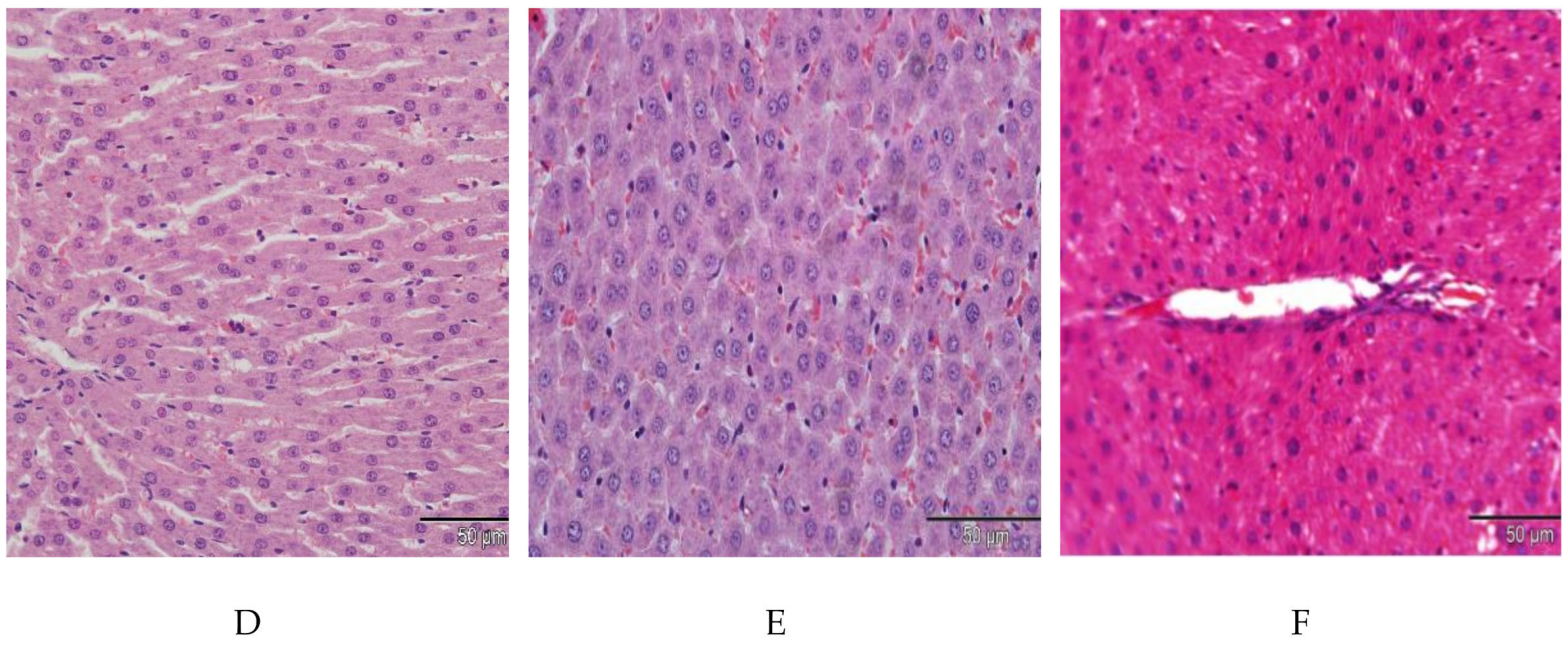
Histopathological analysis of liver (scale bar 50 µm) for the (**A**) NC group, (**B**) PC group, (**C**) DC group, (**D**) T I group, (**E**) T II group, and (**F**) T III group. NC: normal control; PC: positive control; DC: drug control; T I: treatment 1 (10 mg·kg^−1^); T II: treatment 2 (100 mg·kg^−1^); T III: treatment 3 (200 mg·kg^−1^).

In the liver histopathology of the NC group, we observed the normal standard of hepatocytes and sinusoids (Figure 2A). In the PC group, we observed the presence of lipid droplets in the hepatocytes (Figure 2B). Furthermore, there was a severe degree of fat change in the DC group (Figure 2C). The normal strands of hepatocytes were observed in all *E. tapos* shell-treated groups T I, TII, and TIII (Figure 2D–F).

### 2.8. Effect of E. tapos Shell Extract on Histopathology of Adipose Tissue

**Figure 3:**
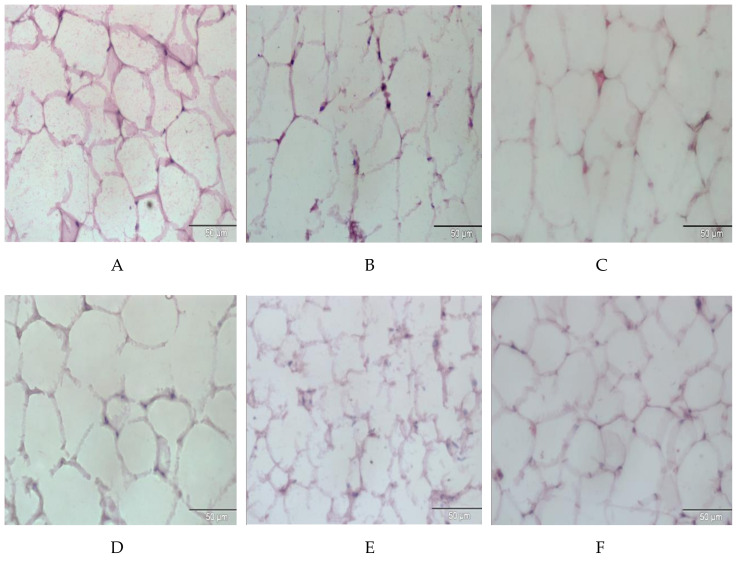
Histopathological analysis of RpWAT (scale bar 50 µm) for the (**A**) NC group, (**B**) PC group, (**C**) DC group, (**D**) T I group, (**E**) T II group, and (**F**) T III group. NC: normal control; PC: positive control; DC: drug control; T I: treatment 1 (10 mg·kg^−1^); T II: treatment 2 (100 mg·kg^−1^); T III: treatment 3 (200 mg·kg^−1^).

The histopathology of adipose tissue in the NC group indicated a normal size of adipocytes as compared to the PC group (Figure 3A). In PC, DC, and T1 groups, hypertrophy of adipocytes was observed (Figure 3B–D). In the *E. tapos* shell-treated groups, doses of 100 mg/kg (T II) and 200 mg/kg (T III) resulted in normal size of adipocytes comparable to the NC group (Figure 3E,F).

## 3. Discussion

HF and CAF diet consisting ingredients consumed in our daily life were used to induce obesity. According to the results, the HF and CAF diet-induced rats significantly gained weight as compared to rats fed with standard chow diet. These findings were similar to a previous study that showed body weight gain due to hyperphagia in rats [10]. Both energy-dense and palatable diets introduced to the rats contributed to fat hyperphagia and reduced sensitivity to peptide satiety, which resulted in overeating leading to obesity [11]. Furthermore, consumption of HF diet increased the caloric intake and caused excess body weight in humans and animals [12]. Orlistat being readily available in the market demonstrates adverse side effects such as subacute liver failure, diarrhea, and severe hepatic injury [13].

With such consequences, natural resources have been used for obesity treatment on top of having lower toxicity, they also have fewer side effects, as compared to antiobesity drugs [5,10]. In the current study, we analyzed the anti-obesity activity of *E. tapos* shell extract in obesity-induced Sprague Dawley rats. The *E. tapos* shell extract prepared via hot aqueous extraction was found to have a higher flavonoid content and antioxidant activity when compared to *E. tapos* seed extract [14]. Moreover, a previous study revealed that *E. tapos* shell extract generally has higher pancreatic lipase, α-amylase, and α-glucosidase inhibitory effects than *E. tapos* seed [15]. In this study, the result showed that *E. tapos* shell extract had a high flavonoid content with the highest peak (9 × 10^7^ counts) observed in the LC–MS analysis (Figure 1). Flavonoids have the potential in regulating digestion of carbohydrates and the deposition of adipose, which helps in combating obesity. 

Rats supplemented with *E. tapos* shell extract had significantly lower body weights. The reduction in body weight could be due to decrease in food intake or higher energy expenditure [16]. The outcome from the group supplemented with *E. tapos* shell extract had no significant difference in terms of caloric intake compared to those of the PC group. Result suggests that the reduction in body weight were not solely dependent on the reduction in calorie intake. This is further supported by Karimi et al. [17], who claimed that increased energy spending and alterations of the intestinal barriers were what caused the inhibition of body weight gain.

Additionally, the high content of flavonoids in *E. tapos* shell contributed to the slight inhibition of plasma lipids for the obese model. A slight reduction of TC and LDL may be contributed by the flavonoids present in *E. tapos* shells, which posed as an inhibitor of lipid oxidation, thus facilitating lipolysis. A similar study by Koshy et al. [18] demonstrated that the intake of flavonoids from *Garcinia cambogia* significantly lowered lipid levels in high-cholesterol diet-fed rats. A significant reduction in plasma TG alongside with the improvement in liver steatosis were observed in a study on obese mice supplemented with flavonoid-rich peels of the *Citrus unshiu* fruit for 42 days [19].

Inhibiting the metabolism of triglyceride is one of a therapeutic approach for obesity. Excess triglycerides are stored in adipocytes as large lipid droplets. Meanwhile, in the liver, the PC group expressed the highest TG level among all groups, in accordance with Klop et al. [20], that the accumulation of TG was present in the liver of their obese model was higher due to impaired lipid hydrolysis, and increased fluxes of free fatty acid to the liver. The potential of flavonoids in the treatment modality of obesity has been reported in numerous studies [21]. A study by Miwa et al. [22] revealed that patients with hypertriglyceridemia had distinct reduction in plasma TG level when consumed flavonoid glucosyl hesperidin (500 mg/day) for 6 months. The study stated that quercetin, a flavonoid, aided the breakdown of lipid in adipose tissues, thus triggering weight loss by boosting blood circulation to the muscles [22].

Lipoprotein lipase (LPL) is an enzyme that plays a pivotal role in the catabolism of TG in adipose tissue and circulation. In adipose tissue, LPL acts as a “gate” for the entrance and reconversion of free fatty acids. Some inhibition of the LPL activity was noted in this study, which could be attributed to the various known phytochemicals in *E. tapos* shell extract. The phenolic compounds found in the shell extract might contribute to the hypolipidemic properties of *E. tapos.* The supplementation of polyphenols in animal models strongly demonstrated a prominent anti-obesity effect, as reflected by the loss of body weight. Another study by Yao et al. [23] reported that serum LPL level was significantly reduced upon the administration of apple polyphenol extract for 1 week.

Adipose is the main energy-storing depot, which has an infinite ability to multiply with chronic exposure to an energy-dense diet, thereby eventually promoting metabolic syndromes, particularly obesity [24]. *E. tapos* shell extract supplementation showed a high potency in suppressing the accumulation of fat in white adipose tissues, as well as visceral fat. Obesity is often accompanied by liver steatosis and cirrhosis, hallmarks of liver diseases. The mass of lipids in healthy human liver may add up to 5% of its weight [25]. On the other hand, liver weight in obese Sprague Dawley rats was significantly increased in the study conducted by D’souza et al. [26], similar to our study. The increase in liver mass corresponds with the event of fat accumulation in hepatocytes, commonly known as steatosis [26]. A previous in vivo study using mulberry water extracts containing a high level of polyphenols demonstrated decreased hepatic lipids in an HF diet model [27].

However, the excessive uptake of fatty acid is what leads to the progress of steatosis. Additionally, obesity is typically characterized by a surplus mass of adipose tissues and enlarged adipocyte size. The expansion of adipocytes caused by excessive fatty acid deposition as a results of prolonged feeding of HF diets [28]. MacLean et al. mentioned that weight reduction is accompanied by decreases in the size of adipocytes [29]. Our findings were consistent with the study conducted by Marques et al. [30], who showed that the size of adipocytes was significantly larger in obese rats as compared to the normal group. Our outcomes demonstrate that the size of adipocytes in all *E. tapos* shell extract-supplemented groups were significantly smaller than that in the obese control group. Here, we postulate that the *E. tapos* shell extract possesses potential anti-obesity bioactive compounds that promote the inhibition of adipogenesis.

## 4. Materials and Methods

### 4.1. Sample, Diets, Chemicals, and Biochemical Analysis

#### 4.1.1. Preparation of *E. tapos* Shell Extraction

Fresh fruits of *E. tapos* were collected from Pahang, Malaysia. The plant sample was sent to the Institute of Bioscience, Universiti Putra Malaysia (UPM) for identification (Voucher number: SK3154/17). They were cleaned and oven-dried at 60 °C for 24 h. The outer shell was separated from the seed, and it was ground to produce a powdered sample. *E. tapos* extraction was carried out using a hot aqueous extraction method. The ground sample of *E. tapos* shell weighing 50 g was extracted in 500 mL of 70 °C distilled water for 24 h, whereas the dilution was based on the standard solvent-to-sample ratio of extraction preparation which was 1:10. The *E. tapos* aqueous extract was then filtered, freeze-dried, and stored at −20 °C. The *E. tapos* shell extract was analyzed by LC–MS (liquid chromatography–tandem mass spectrometry) to identify potential bioactive compounds [31].

#### 4.1.2. Preparation of High-Fat (HF) Diet

The HF diet composition was adapted from Levin and Dunn-Meynell [32]. The HF diet contains 414.0 kcal/100 g with carbohydrate (43%), protein (17%), and fat (40%). The diet is composed of 50% commercial food pellet, 20% milk powder (Dutch Lady), 24% ghee (Crispo), and 6% corn starch. Meanwhile, the standard rat pellet contains 306.2 kcal/100 g with 48.8% carbohydrate, 21% protein, and 3% fat. All these ingredients were properly mixed and baked in the oven at 60 °C for 2 h.

#### 4.1.3. Biochemical Analysis

Diagnostic reagent test kits were purchased from ADVIA Chemistry (Tarrytown, NY, USA) to examine the total cholesterol (TC), low-density lipoprotein (LDL), and high-density lipoprotein (HDL) levels. BioAssays System Triglycerides Assay Kit was used to analyze triglycerides (TG). Lipoprotein lipase (LPL) activity was analyzed using an LPL assay kit from Biovision (Milpitas, CA, USA). LC–MS analysis was used to identify phytochemicals in the aqueous shell extracts of *E. tapos.*

### 4.2. Animals

Thirty-six male Sprague Dawley (SD) rats (150 g) were housed at 28 ± 2 °C and maintained on a 12:12 h light/dark cycle. All procedures involving animals were approved by the Animal Care and Ethics Committee, UPM, Malaysia (IACUC R037/2016).

### 4.3. Measurement of Growth Indicators

Body weight and food intake were measured weekly.

### 4.4. Experimental Design

After 1 week of acclimatization, the rats were divided into two groups.

Group 1: Normal control (NC) rats were fed with standard chow diet (11 kJ/kg, 14% fat, 21% protein, 65% carbohydrate by energy). Remaining rats were fed with an HF diet (1732 kJ/100 g) and CAF diet (chicken roll (987 kJ/100 g), salty biscuit (2292 kJ/100 g), cake (2033 kJ/100 g), cheese snacks (2322 kJ/100 g)) to generate obesity for 4 weeks. After four weeks of obesity induction, the obese rats were further divided into five groups as described below.

Group 2: Positive control (PC) no treatment given. 

Group 3: Drug control (DC) rats received 10 mg/kg of body weight of Orlistat. 

Group 4: Treatment 1 (T I) rats orally received *E. tapos* shell extract (10 mg/kg of body weight) daily for 4 weeks. 

Group 5: Treatment 2 (T II) rats orally received *E. tapos* shell extract (100 mg/kg of body weight) daily for 4 weeks. 

Group 6: Treatment 3 (T III) rats orally received *E. tapos* shell extract (200 mg/kg of body weight) daily for 4 weeks.

### 4.5. Collection of Specimens

At the end of the study, all rats were anesthetized, and blood was collected via cardiac puncture. The organs such as liver and white adipose tissues were collected for further analysis.

### 4.6. Histopathological Assessment of Liver and Adipose Tissue

A section of the upper lobe of the liver and retroperitoneal white adipose tissue (RpWAT) was dissected out and placed in 10% formaldehyde for a week, then it was embedded in paraffin wax. The block of the embedded tissue was cut into 4 to 7 µm thick sections. The sectioned tissue sample was placed on slides and was stained with hematoxylin and eosin (H&E). The slides were examined under a light microscope [10].

### 4.7. Statistical Analysis

Statistical analyses were performed using SPSS software version 23 (SPSS Inc., Chicago, IL, USA). The results were interpreted as the mean ± standard deviation (SD). All data were tested for normality. Body weight, caloric intake, and biochemical analysis were analyzed using one-way ANOVA followed by a post hoc least significant difference (LSD) test, and the differences were considered statistically significant at the level of *p* < 0.05.

## 5. Conclusions

In conclusion, supplementation with *E. tapos* shell extract in obesity-induced rats indicates the potential for body weight reduction, a positive lipid-lowering effect, decrements in triglyceride accumulation and LPL activity, and an improvement in the histopathology of the liver, as well as adipose tissues. The findings of this study revealed that *E. tapos* shell extract at a dose of 200 mg/kg showed the most effective result compared to 10 mg/kg and 100 mg/kg doses.

## Figures and Tables

**Table 1:** Anti-obesity effect of *E.tapos* shell extract on body weight and caloric intake.

Group	Body Weight at Death (g)	Caloric Intake (kJ)
NC	292.7 ± 37.7 ^b^	5890.5 ± 3554.5 ^b^
PC	350.0 ± 15.0 ^a^	9341.9 ± 781.52 ^a^
DC	310.0 ± 24.5 ^b^	8653.6 ± 834.32
T I	292.2 ± 41.6 ^b^	7578.0 ± 983.45 ^ab^
T II	299.2 ± 44.1 ^ab^	8064.8 ± 914.01 ^ab^
T III	288.0 ± 42.8 ^b^	7971.6 ± 945.46 ^a^

Results are presented as mean ± SD. ^a^ indicates *p* < 0.05 compared with NC group, and ^b^ indicates *p* < 0.05 compared with PC group. NC: normal control; PC: positive control; DC: drug control; T I: treatment 1 (10 mg·kg^−1^); T II: treatment 2 (100 mg·kg^−1^); T III: treatment 3 (200 mg·kg^−1^).

**Table 2:** Anti-obesity effect of *E. tapos* shell extract on organ weight.

Group	Organ Weight (g)			
	Liver (g)	RpWAT (g)	Visceral Fat (g)	Gonadal Fat (g)
NC	9.7 ± 1.5 ^ab^	2.2 ± 0.6 ^b^	2.1 ± 0.4 ^b^	2.7 ± 0.7 ^b^
PC	11.7 ± 1.7 ^a^	4.7 ± 1.0 ^a^	3.3 ± 0.4 ^a^	4.2 ± 1.0 ^a^
DC	9.0 ± 1.4 ^b^	3.1 ± 0.6 ^b^	2.4 ± 0.5 ^b^	3.5 ± 0.7 ^ab^
T I	9.2 ± 2.0 ^b^	3.3 ± 1.6 ^b^	2.1 ± 0.6 ^b^	3.3 ± 1.0 ^ab^
T II	8.9 ± 1.7 ^b^	3.9 ± 1.3 ^a^	2.6 ± 0.9 ^ab^	3.0 ± 0.5 ^b^
T III	9.0 ± 2.6 ^b^	4.3 ± 1.3 ^a^	2.8 ± 0.9 ^a^	3.6 ± 0.6 ^ab^

Results are presented as mean ± SD. ^a^ indicates *p* < 0.05 compared with NC group, and ^b^ indicates *p* < 0.05 compared with PC group. NC: normal control; PC: positive control; DC: drug control; T I: treatment 1 (10 mg·kg^−1^); T II: treatment 2 (100 mg·kg^−1^); T III: treatment 3 (200 mg·kg^−1^).

**Table 3:** Anti-obesity effect of *E. tapos* shell extract on lipid profiles.

Group	Lipid Profile (mmol/L)		
	TC	LDL	HDL
NC	1.38 ± 0.26	0.76 ± 0.28 ^b^	0.39 ± 0.08
PC	1.63 ± 0.33	1.04 ± 0.16 ^a^	0.35 ± 0.05
DC	1.58 ± 0.29	0.98 ± 0.26 ^ab^	0.43 ± 0.08
T I	1.58 ± 0.16	0.88 ± 0.21 ^ab^	0.42 ± 0.10
T II	1.49 ± 0.17	0.88 ± 0.18 ^ab^	0.40 ± 0.07
T III	1.48 ± 0.16	0.86 ± 0.20 ^ab^	0.41 ± 0.08

Results are presented as mean ± SD. ^a^ indicates *p* < 0.05 compared with NC group, and ^b^ indicates *p* < 0.05 compared with PC group. NC: normal control; PC: positive control; DC: drug control; T I: treatment 1 (10 mg·kg^−1^); T II: treatment 2 (100 mg·kg^−1^); T III: treatment 3 (200 mg·kg^−1^). TC, total cholesterol; LDL, low-density lipoprotein; HDL, high-density lipoprotein.

**Table 4:** Anti-obesity effect of *E. tapos* shell extract on triglycerides.

Group	Triglycerides (mmol/L)		
	Plasma	RpWAT	Liver
NC	1.25 ± 0.20 ^b^	16.04 ± 1.82 ^b^	20.82 ± 8.05
PC	1.77 ± 0.84 ^a^	26.26 ± 14.29 ^a^	28.39 ± 8.97
DC	1.40 ± 0.33 ^ab^	20.69 ± 7.27 ^ab^	23.59 ± 8.20
T I	1.33 ± 0.21 ^ab^	19.70 ± 3.41 ^ab^	27.11 ± 11.07
T II	1.25 ± 0.23 ^ab^	18.86 ± 1.87 ^ab^	23.13 ± 4.77
T III	1.32 ± 0.23 ^ab^	19.62 ± 3.67 ^ab^	24.29 ± 4.96

Results are presented as mean ± SD. ^a^ indicates *p* < 0.05 compared with NC group, and ^b^ indicates *p* < 0.05 compared with PC group. NC: normal control; PC: positive control; DC: drug control; T I: treatment 1 (10 mg·kg^−1^); T II: treatment 2 (100 mg·kg^−1^); T III: treatment 3 (200 mg·kg^−1^). RpWAT, retroperitoneal white adipose tissue.

**Table 5:** Anti-obesity effect of *E. tapos* shell extract on lipoprotein lipase (LPL) activity.

Group	Lipoprotein Lipase	
	Plasma (mU/mL)	RpWAT (mU/g)
NC	1.25 ± 0.20 ^b^	16.04 ± 1.82 ^b^
PC	1.77 ± 0.84 ^a^	26.26 ± 14.29 ^a^
DC	1.40 ± 0.33 ^ab^	20.69 ± 7.27 ^ab^
T I	1.33 ± 0.21 ^ab^	19.70 ± 3.41 ^ab^
T II	1.25 ± 0.23 ^ab^	18.86 ± 1.87 ^ab^
T III	1.32 ± 0.23 ^ab^	19.62 ± 3.67 ^ab^

Results are presented as mean ± SD. ^a^ indicates *p* < 0.05 compared with NC group, and ^b^ indicates *p* < 0.05 compared with PC group. NC: normal control; PC: positive control; DC: drug control; T I: treatment 1 (10 mg·kg^−1^); T II: treatment 2 (100 mg·kg^−1^); T III: treatment 3 (200 mg·kg^−1^).

**Table 6:** Bioactive compound identified from *E. tapos* shell extract by LC-MS.

Name of the Compound	Retention Time (min)	Mass (g/mol)
3′4′5-trimethoxyflavone (Flavonoid)	4.910	188.116
Acetyl lysine	5.987	282.1681
7-methoxychromone	10.165	176.0476
C11 H19 N O2	13.845	197.1419
C20 H30 N4 O7 S	18.503	470.1832
C23 H44 N4 O4	21.636	440.3363
Undulatone	26.527	534.2099
Aldosterone 18-glucuronide	28.334	536.2261
